# Methicillin-Resistant *Staphylococcus aureus* Infections in Human Immunodeficiency Virus-Infected Children and Adolescents

**DOI:** 10.1155/2012/627974

**Published:** 2012-09-12

**Authors:** George K. Siberry, Toni Frederick, Patricia Emmanuel, Mary E. Paul, Beverly Bohannon, Travis Wheeling, Theresa Barton, Mobeen H. Rathore, Kenneth L. Dominguez

**Affiliations:** ^1^Pediatric, Adolescent & Maternal AIDS (PAMA) Branch, Eunice Kennedy Shriver National Institute of Child Health & Human Development, National Institutes of Health, Bethesda, MD 20892-7510, USA; ^2^Maternal, Child, Adolescent/Adult Center for Infectious Diseases and Virology, University of Southern California, Los Angeles, CA 90033, USA; ^3^Department of Pediatrics, University of South Florida College of Medicine, Tampa, FL 33606, USA; ^4^Department of Pediatrics, Baylor College of Medicine, Houston, TX 77030, USA; ^5^Division of HIV/AIDS Prevention, Centers for Disease Control and Prevention, Atlanta, GA 30333, USA; ^6^Northrop Grumman Inc., Atlanta, GA 30345, USA; ^7^Department of Pediatric Infectious Diseases, University of Texas Southwestern Medical Center, Dallas, TX 75390, USA; ^8^Department of Pediatric Infectious Diseases and Immunology, University of Florida and Wolfson Children's Hospital, and University of Florida Center for HIV/AIDS Research, Education and Service (UF CARES), Jacksonville, FL 32209, USA

## Abstract

*Background*. Methicillin-resistant *Staphylococcus aureus* (MRSA) infection incidence has increased in healthy US children. Our objective was to evaluate MRSA incidence and correlates in HIV-infected youth. *Methods*. The CDC-sponsored LEGACY study is a US multicenter chart abstraction study of HIV-infected youth. We identified MRSA infections among participants with ≥1 visit during 2006. We used bivariate and multivariable analyses to compare sociodemographic and HIV clinical factors between MRSA cases and noncases. *Results*. Fourteen MRSA infections (1 invasive, 12 soft tissue, 1 indeterminate) occurred among 1,813 subjects (11.1 infections/1,000 patient-years (PY), 95% CI: 11.06–11.14). Most (86%) isolates were clindamycin susceptible. Compared with noncases, MRSA cases were more likely older (17 versus 14 years), black (100% versus 69%), behaviorally HIV infected (43% versus 17%), and in Maryland (43% versus 7%) and had viral loads (VL) >1000 copies/mL (86% versus 51%) and lower mean CD4% (18% versus 27%) (all *P* < 0.05). In multivariate analysis, independent risk factors were Maryland care site (adjusted odds ratio (aOR) = 9.0), VL >1000 copies/mL (aOR = 5.9), and black race (aOR undefined). *Conclusions.* MRSA occurred at a rate of 11.1 infections/1,000 PY in HIV-infected youth but invasive disease was uncommon. Geographic location, black race, and increased VL, but not immunosuppression, were independently associated with MRSA risk.

## 1. Introduction

Throughout the United States, MRSA infections have increased dramatically in healthy adults and children without more commonly healthcare-associated MRSA (HA-MRSA) risk factors, such as recent hospitalization or surgery, indwelling catheter or residence in a long-term care facility [1–5]. Estimated rates of community-associated MRSA (CA-MRSA) infection are 18–26 per 100,000 persons annually with higher rates in young children and blacks [3]. Most circulating CA-MRSA strains cause skin and soft tissue infections (SSTIs), though invasive infections occur and may be increasing [6–9]. These same CA-MRSA strains have also increasingly caused infections in healthcare settings and in patients with traditional risk factors for HA-MRSA [10–12].

HIV infection appears to be an independent risk factor for MRSA infections in adults, and MRSA infections occur at higher rates in HIV-infected adults [13, 14]. The association of lower CD4+ T-lymphocyte (CD4) counts with higher risk of MRSA infection suggests that the MRSA infection risk may be increased by immunodeficiency [13] and raises concern for higher risk of invasive MRSA infection. However, low CD4 count is not a consistent risk factor [14, 15], and the vast majority of MRSA infections are SSTIs [13]. Several studies suggest that concurrent behavioral and psychosocial risk factors, such as high-risk sexual activity, illicit drug use, and poor hygiene, play a substantial role in the increased MRSA risk in HIV-infected adults [13–17].

Little is known about the epidemiology of MRSA infections in HIV-infected children and adolescents [18], who, like HIV-infected adults, have high rates of immunosuppression, antibiotic use, intravascular catheter use, and healthcare contact, all of which may increase risk of MRSA infection. However, the behavioral and psychosocial risk factors for MRSA infection in HIV-infected adults may be less common in HIV-infected children and adolescents. In addition, rates of MRSA infections in HIV-infected children may reflect the risk of pediatric CA-MRSA infections in healthy children in their communities. The objective of this study was to estimate the incidence of MRSA infections in a national sample of HIV-infected children and adolescents, to characterize the nature and severity of MRSA infections in this population and to explore risk factors for MRSA infection. 

## 2. Patients and Methods

### 2.1. LEGACY Study

The Longitudinal Epidemiologic Study to Gain Insight into HIV/AIDS in Children and Youth (LEGACY) study is a CDC-funded, observational, prospective cohort study of HIV-infected children, and adolescents enrolled between birth and 24 years of age from 22 HIV specialty centers across the US. This study population was selected using a 3-stage cluster probability proportional to size sampling method to encourage a broad selection of HIV-infected infants, children and adolescents receiving care in geographically diverse small, intermediate, and large-sized facilities. This study was approved by the Institutional Review Board (IRB) of the CDC and the IRBs of all local study sites. A consolidated 301(d) Certificate of Confidentiality was obtained for LEGACY to provide an added level of strict privacy protection for participants. Between November 2005 and June 2007, at least 80% of eligible HIV-infected youth presenting for care at LEGACY clinic sites were offered enrollment. Participation was voluntary. Written informed assent and consent were obtained from minors and parents, as appropriate. The medical records of participants were reviewed, and data recorded on all visits during the study period were abstracted by trained data abstractors. Data collected included demographics (i.e., age, race, ethnicity, gender, educational level); mode of HIV infection (i.e., perinatal, breastfeeding, heterosexual activity, homosexual activity, sexual abuse, blood transfusion and injection drug use (IDU)); clinical diagnoses; antiretroviral (ARV) and non-ARV medications; immunizations; laboratory test results, including CD4 cell counts, plasma HIV RNA viral loads, results of HIV genotypic and phenotypic ARV resistance tests, chemistries, hepatitis testing, and urinalyses; reproductive history (i.e., age of menarche, sexual activity, contraceptive use, history of previous pregnancies and diagnosed sexually transmitted infections); psychosocial data (i.e., HIV disclosure status, substance abuse, sexual history, caregiver data); mortality data. Highly active antiretroviral therapy (HAART) was defined as treatment with at least three ARV drugs at the same time.

### 2.2. MRSA Analysis

This analysis included data on participants with clinical and laboratory data available from at least one visit in 2006. A case of MRSA infection was defined as an individual who had any mention of MRSA as either a reason for hospitalization, as a diagnosis listed in a medical visit, or as the organism isolated from a culture at any visit in 2006. Participants with no mention of MRSA at any 2006 visit were considered as not having MRSA (MRSA noncases). Person-time of observation was calculated using January 1, 2006 as the study start date for individuals with a visit before 1/1/06 or the first visit in 2006 if they did not have a visit before 1/1/06. Time to event for MRSA cases was censored at the date of their MRSA infection. Time to event for non-MRSA cases was censored either at 12/31/06 for those with a visit after 12/31/06 or at their last visit in 2006 if they were not followed into 2007. For MRSA cases, age was calculated as the age at MRSA diagnosis. CD4 cell count and percentage, HIV RNA viral load (viral load), HAART use, and absolute neutrophil count (ANC) were defined as the closest value before MRSA diagnosis. For the MRSA noncases, age, CD4 cell count, viral load, HAART use (yes/no), and ANC were determined at the first visit in 2006. Mode of HIV transmission was defined as (i) vertical (mother-to-child) or perinatal, (ii) behavioral, or (iii) other or unknown. Behavioral mode of HIV transmission included injection drug use and sexual exposure. Demographic, psychosocial, and HIV clinical factors were examined as predictors of risk for MRSA infection using the chi-square test and Fisher's exact test for categorical variables and the *t*-test for continuous variables. Risk factors for which the odds ratio was statistically significant (*P* < 0.05) on bivariate analysis were included in a multivariable logistic regression to determine which factors remained independently associated with MRSA infection after controlling for confounding. Data were analyzed using SAS version 9.2.

## 3. Results

Clinical and laboratory data were available from 1,813 subjects with clinical and laboratory data available from at least one visit in 2006 from 16 sites. Fourteen MRSA infections (all culture confirmed) occurred among 1,813 subjects and 1,267 person-years of followup for an incidence of 772 cases per 100,000 persons and an incidence rate of 11.1 infections per 1,000 patient-years (PY) of observation (95% confidence interval (CI): 11.06–11.14). Median follow-up time was 0.79 years for the MRSA-infected case-patients and 0.80 years for the MRSA-uninfected patients. Seven (0.45%) subjects who were perinatally infected with HIV had MRSA infections for an incidence of 6.8 infections per 1,000 PY (95% CI, 1.8–11.8). Six (1.9%) behaviorally HIV-infected subjects had an MRSA infection incidence of 33.3 infections per 1,000 PY (95% CI, 5.6–61). One MRSA-infected case-patient acquired HIV infection in childhood but HIV risk factors were not known; this child was classified as nonbehaviorally HIV infected. In most cases (11/14), MRSA was diagnosed and/or cultured at a visit in 2006 after at least one prior visit without MRSA infection. In the remaining three cases, MRSA was diagnosed and/or cultured at the first abstracted visit for LEGACY, in February, April, and July, respectively. 

All MRSA-infected case-patients were non-Hispanic black; eight (57%) were female and six (43%) received HIV care in Maryland. None reported use of intravenous drugs. Median age was 18 years (range, 6–24). Median CD4 cell count and percentage were 383 cells/mm^3^ and 20%, respectively. Six (43%) subjects were treated with HAART, but only 1 (7%) had a suppressed viral load (<400 copies/mL). Overall, nine (64%) MRSA-infected case-patients had HIV-1 viral loads >10,000 copies/mL. One subject was receiving cotrimoxazole prophylaxis; none were taking systemic steroids.

Four (29%) case-patients were hospitalized at time of MRSA diagnosis, including one subject whose blood culture grew MRSA and whose urine culture grew *E. coli* and MRSA. This subject with invasive disease was a 15-year-old, perinatally HIV-infected boy who was taking HAART; the laboratory results from five months prior to onset of his MRSA infection included CD4 values of 524 cells/mm^3^ and 28% but a viral load of 49,411 copies/mL. He did not have an indwelling vascular catheter or neutropenia at the time of hospital admission for his MRSA infection. There was no evidence of endocarditis, and he responded well to intravenous treatment for urinary tract infection with bacteremia. Another subject with severe, chronic cystic lung disease who was admitted for management of a recurrent pneumothorax produced purulent sputum with heavy normal respiratory flora noted on Gram stain and growth of MRSA (at 2 days), *Pseudomonas aeruginosa*, and normal respiratory flora. Sputum production and cough improved with piperacillin/tazobactam and tobramycin but without MRSA-active antibiotic treatment. The role of MRSA in this exacerbation of chronic pulmonary disease was uncertain. There were no other cases of bacteremia or other invasive infections. MRSA was cultured from skin and soft tissue infections (SSTIs) in the remaining 12 (71%) case-patients. Two of these SSTIs were associated with viral exanthems (zoster and HSV). All MRSA isolates were susceptible to vancomycin and cotrimoxazole; resistance to erythromycin (11/12 tested) was more common than resistance to clindamycin (one constitutive resistance and one inducible resistance, both from Maryland, detected among 14 tested). Most isolates were susceptible to tetracycline (9/11 tested) and to fluoroquinolones (6/8 tested).

Compared with MRSA-uninfected participants, MRSA case-patients were older (17 versus 14 years, *P* < 0.05), more likely to be non-Hispanic black (100% versus 69%, *P* = 0.012), to have received care in Maryland (43% versus 7%, *P* < 0.0001), and to have been behaviorally HIV infected (43% versus 17%, *P* < 0.012) (Table 1). MRSA case-patients were also more likely to have had an HIV-1 viral load > 1000 (86% versus 51%, *P* = 0.01), a lower mean CD4 percentage (18% versus 27%, *P* = 0.0016) and a lower mean CD4 count (391 versus 680 cells/mm^3^, *P* = 0.029). MRSA-infected and MRSA-uninfected subjects were similar (*P* > 0.05) with respect to gender (57% versus 55% female), history of eczema (14% versus 13%), presence of ANC < 1500 cells/mm^3^ (30% versus 27%), history of AIDS-defining illness (18% versus 25%), and HAART use (43% versus 45%). 

In multivariate analysis (Table 2), Maryland care site, non-Hispanic black race, and HIV-1 viral load >1000 copies/mL remained independently associated with MRSA infection after controlling for age, behavioral mode of HIV transmission, and CD4 count. Because CD4 count and viral load were highly correlated, we tested the model without viral load; the association with CD4 count remained nonsignificant. In the final reduced model, Maryland HIV care site, non-Hispanic black race, and HIV-1 viral load > 1000 copies/mL remained independently associated with MRSA infection. 

## 4. Discussion

This analysis provides the first estimate of annual incidence of MRSA infections in a well-defined cohort of children and adolescents with HIV infection since the emergence of the CA-MRSA epidemic. The predominance of SSTIs, the low rate of bacteremia or other invasive infections, and the very low rate of clindamycin resistance are more typical of CA-MRSA infections and suggest that most of these infections were caused by CA-MRSA rather than HA-MRSA strains [1, 5, 19]. The incidence rate in the present study, of 11.1 per 1000 PY, appears to be lower than that of 40.3 per 1,000 PY observed in adult HIV-infected populations [13]. On the other hand, the incidence of 772 per 100,000 children in 2006 in this study was approximately 30-to-50 fold higher than the annual CA-MRSA incidence for 2–18-year-old black children identified from MRSA infection population-based sentinel surveillance in Baltimore (15 per 100,000 children) and Atlanta (25 per 100,000 children) (estimated from Figure 1 in Fridkin 2005) [3]. These data suggest that there is an elevated incidence of MRSA infection in HIV-infected children compared to HIV-uninfected children in population-based sentinel surveillance, just as is seen in HIV-infected adults compared to their HIV-uninfected counterparts. 

Two of the independent risk factors for MRSA infection—black race and Maryland site—observed in this analysis may reflect the higher rates of MRSA infection in blacks and geographic variability in MRSA rates in communities across the United States [3]. Higher viral load, lower CD4 values, older age, and behavioral route of HIV infection were all associated with higher risk of MRSA infection in bivariate analyses, but only higher HIV-1 viral load persisted as a significant risk factor in the multivariate model. These results suggest that poor viral suppression may confer greater risk for MRSA infection than HIV-related immunosuppression or behavioral risk factors. Low CD4 count and high-risk sexual activity, but not HIV-1 viral load, were stronger risk factors in an adult cohort [13]. The rate of high-risk sexual activity overall is greater in an adult cohort, but there is no clear explanation for the differences in predictive value of the HIV-specific indicators between this pediatric cohort and the adult cohort.

While HIV-infected children in this study had a higher rate of MRSA, the spectrum and severity of infections were remarkably similar to the pattern observed in otherwise healthy children [1]. The increased risk in HIV-infected children may be attributable, in part, to greater MRSA exposure because the pediatric HIV epidemic in the US has disproportionately impacted the same urban communities of color where the CA-MRSA epidemic has been most intense. Others have hypothesized HIV-related immunosuppression as the basis of increased MRSA risk in HIV-infected people. However, neutrophils, other aspects of innate immunity not affected by HIV infection, and intact skin provide more important lines of defense against staphylococcal infections than cellular immunity, which may explain the lack of an independent association between low CD4 count and risk of MRSA infection observed in this study. 

 It is not known whether HIV-associated changes in innate immunity increase MRSA risk. Abnormal chemotaxis and phagocytosis have been documented in HIV infection [20] and may mediate increased MRSA risk. Eczema, neutropenia, and steroid use (which impairs neutrophil function) were not apparent mediators of MRSA risk in the present analysis. Two MRSA cases were associated with viral exanthems (HSV and zoster). These exanthems occur at increased rates among HIV-infected persons and compromise skin integrity, which could increase MRSA SSTI risk. However, the low rate of severe MRSA infections in this study is more consistent with greater risk of exposure to MRSA and the intrinsic ability of MRSA organisms circulating in the community to cause SSTIs in all hosts rather than immunodeficiency as the principal mediator of increased MRSA infection risk in HIV-infected children and adolescents. 

This study is limited by its reliance on data abstracted from existing medical records and the lack of availability of bacterial isolates for strain typing to confirm similarity to CA-MRSA strains. The risk of recurrent infections was not evaluable because of incomplete data about MRSA infections before and after 2006. The CD4 count used for each case was the value obtained closest to the time of MRSA diagnosis. Most (9/14) CD4 counts were obtained within 3 months prior to MRSA and all but two within the prior 6 months, so the CD4 values used are expected to accurately reflect the CD4 count at the time onset of MRSA infection. Estimates of MRSA infection rates in this HIV-infected cohort are extrapolated from a small number of events and may be higher than published estimates for healthy children, because HIV-infected children may have more frequent medical care appointments, are often followed by infectious disease specialists, and may be more intensively evaluated for suspected infections. Because we did not have a control group of HIV-uninfected children and did not exclude children based on contact with healthcare in this study, we cannot conclude that HIV infection rather than other risk factors such as poverty, contact with the health care system, or other factors may have played a more significant role. Finally, differences in MRSA rates by geographic location may represent differences in routine practices for evaluation of soft tissue and other infections such as the culturing of purulent drainage from soft tissue abscesses as part of routine management.

## 5. Conclusion

MRSA infections in a cohort of HIV-infected children and adolescents occurred at a rate higher than that observed among healthy children in the general population, but invasive disease was uncommon. Susceptibility profiles of MRSA isolates indicated that most infections were caused by CA-MRSA strains. Our analysis suggests that there was an increased incidence of MRSA infection in HIV-infected children compared to the general population, as has been seen in HIV-infected adults. Geographic location, black race and increased VL, but not immunosuppression, were independently associated with MRSA risk. The high prevalence of MRSA in communities most affected by pediatric HIV infection as well as poorly controlled or advanced HIV infection might have contributed to higher rates of MRSA infection in HIV-infected youth.

## Figures and Tables

**Table 1:** Characteristics of Participants with and without MRSA Infections in the LEGACY Study, 2006.

Characteristic	MRSA cases (*N* = 14)	Non-MRSA cases (*N* = 1,799)	Odds ratio	95% CI	*P* value
Female, *n* (%)	8 (57%)	989 (55%)	1.1	0.4, 3.2	0.87
Non-Hispanic black, *n* (%)	14 (100% )	1237 (69%)	1.5	1.4, 1.5	0.012
Behavioral risk factor for HIV acquisition, *n* (%)	6 (43%)	310 (17%) (7 missing)	3.6	1.2, 10.4	0.012
Viral load >1000 copies/mL, *n* (%)	12 (86%)	910 (51%) (16 missing)	5.8	1.3, 25.8	0.01
Not receiving HAART, *n* (%)	8 (57%)	980 (54%)	1.1	0.4, 3.2	0.85
ANC^1 ^<1500, cells/*μ*L, *n* (%)	3 (30%) (4 missing)	464 (27%) (97 missing)	1.1	0.3, 4.5	0.84
Care site, *n* (%)			10.1^2^	3.5, 29.7	<0.0001
Maryland	6 (43%)	124 (7%)
Georgia	2 (14%)	143 (8%)
Florida	3 (21%)	488 (27%)
Washington D.C.	1 (7%)	141 (8%)
Pennsylvania	1 (7%)	99 (6%)
Texas	1 (7%)	206 (11%)
New York	0 (0%)	294 (16%)
Puerto Rico	0 (0%)	87 (5%)
California	0 (0%)	176 (10%)
New Jersey	0 (0%)	41 (2%)
History of eczema, *n* (%)	2 (14%)	236 (13%)	1.1	0.2, 5.0	0.9
Age ≥15 yrs, *n* (%)	10 (71%)	785 (44%)	3.2	1.0, 10.3	0.037
Mean/median age, years	17.1/18.0	13.8/14.0	—	—	<0.05
CD4 count <200, cells/*μ*L, *n* (%)	4 (29%)	185 (11%) (45 missing)	3.4	1.1, 10.9	0.03
CD4 percent <20%, *n*(%)	7 (50%)	414 (24%) (44 missing)	3.2	1.1, 9.3	0.02
Mean/median CD4 count, cells/*μ*L, (%)	390.9/383.0	680.3/574.0	—	—	0.029
Mean/median CD4 %	17.6/19.5	26.9/27.0	—	—	0.0016
History of AIDS-defining illness	2 (18%) (3 missing)	364 (25%) (335 missing)	0.67	0.1, 3.1	0.61

^1^ANC: absolute neutrophil count.

^2^Comparing Maryland versus all others.

MRSA: methicillin-resistant *Staphylococcus aureus.*

**Table 2:** Factors associated with MRSA infection in multivariable analysis, the LEGACY Study, 2006.

	Initial multivariable model (*N* = 1,756)			Final reduced multivariable model (*N* = 1,797)		
Characteristic	aOR	95% CI	*P* value	aOR	95% CI	*P* value
Maryland care site^1^	7.4	2.4, 23.2	0.0006	9.0	3.0, 26.8	<0.0001
CD4 <200 cells/*μ*L^2^	1.6	0.43, 5.7	0.50	—	—	—
Behavioral risk^3^	1.9	0.5, 7.3	0.34	—	—	—
Viral load >1000 copies/mL^4^	4.6	1.0, 22.0	0.056	5.9	1.3, 26.5	0.02
Age ≥15 years^5^	1.4	0.30, 6.1	0.69	—	—	—
Non-Hispanic black^6^	Und^7^	Und^7^	0.035	Und^7^	Und^7^	0.044

Comparing: ^1^Maryland care site versus all other care sites; ^2^CD4 <200 cells/*μ*L versus CD4 ≥200 cells/*μ*L; ^3^Behavioral HIV infection risk versus all other HIV risk categories; ^4^HIV RNA viral load >1000 copies/mL versus viral load ≤1000 copies/mL; ^5^Age ≥15 years versus age <15 years; ^6^Non-Hispanic black versus all other racial/ethnic groups; ^7^Undefined aOR because all MRSA cases were black race.

aOR: adjusted Odds Ratio.

MRSA: methicillin-resistance *Staphylococcus aureus.*

## Abbreviations

MRSA: Methicillin-resistant *Staphylococcus aureus*
HIV: Human immunodeficiency virus.

## References

[1] Dietrich DW, Auld DB, Mermel LA (2004). Community-acquired methicillin-resistant *Staphylococcus aureus* in southern New England children. *Pediatrics*.

[2] Moran GJ, Krishnadasan A, Gorwitz RJ (2006). Methicillin-resistant S. aureus infections among patients in the emergency department. *The New England Journal of Medicine*.

[3] Fridkin SK, Hageman JC, Morrison M (2005). Methicillin-resistant *Staphylococcus aureus* disease in three communities. *The New England Journal of Medicine*.

[4] Gorwitz RJ, Jernigan DB, Powers JH, Jernigan JA http://www.cdc.gov/ncidod/dhqp/ar_mrsa_ca.html.

[5] Chen AE, Goldstein M, Carroll K, Song X, Perl TM, Siberry GK (2006). Evolving epidemiology of pediatric *Staphylococcus aureus* cutaneous infections in a Baltimore hospital. *Pediatric Emergency Care*.

[6] Mongkolrattanothai K, Boyle S, Kahana MD, Daum RS (2003). Severe *Staphylococcus aureus* infections caused by clonally related community-acquired methicillin-susceptible and methicillin-resistant isolates. *Clinical Infectious Diseases*.

[7] Francis JS, Doherty MC, Lopatin U (2005). Severe community-onset pneumonia in healthy adults caused by methicillin-resistant *Staphylococcus aureus* carrying the Panton-Valentine leukocidin genes. *Clinical Infectious Diseases*.

[8] Gonzalez BE, Martinez-Aguilar G, Hulten KG (2005). Severe staphylococcal sepsis in adolescents in the era of community-acquired methicillin-resistant *Staphylococcus aureus*. *Pediatrics*.

[9] Adem PV, Montgomery CP, Husain AN (2005). *Staphylococcus aureus* sepsis and the Waterhouse-Friderichsen syndrome in children. *The New England Journal of Medicine*.

[10] Zaoutis TE, Toltzis P, Chu J (2006). Clinical and molecular epidemiology of community-acquired methicillin-resistant *Staphylococcus aureus* infections among children with risk factors for health care-associated infection 2001–2003. *Pediatric Infectious Disease Journal*.

[11] Hultén KG, Kaplan SL, Gonzalez BE (2006). Three-year surveillance of community onset health care-associated *Staphylococcus aureus* infections in children. *Pediatric Infectious Disease Journal*.

[12] Seybold U, Kourbatova EV, Johnson JG (2006). Emergence of community-associated methicillin-resistant *Staphylococcus aureus* USA300 genotype as a major cause of health care-associated blood stream infections. *Clinical Infectious Diseases*.

[13] Crum-Cianflone NF, Burgi AA, Hale BR (2007). Increasing rates of community-acquired methicillin-resistant *Staphylococcus aureus* infections among HIV-infected persons. *International Journal of STD and AIDS*.

[14] Diep BA, Chambers HF, Graber CJ (2008). Emergence of multidrug-resistant, community-associated, methicillin-resistant *Staphylococcus aureus* clone USA300 in men who have sex with men. *Annals of Internal Medicine*.

[15] Lee NE, Taylor MM, Bancroft E (2005). Risk factors for community-associated methicillin-resistant *Staphylococcus aureus* skin infections among HIV-positive men who have sex with men. *Clinical Infectious Diseases*.

[16] Anderson EJ, Hawkins C, Bolonn MK, Palella FJ (2006). A series of skin and soft tissue infections due to methicillin-resistant *Staphylococcus aureus* in HIV-infected patients. *Journal of Acquired Immune Deficiency Syndromes*.

[17] Senthilkumar A, Kumar S, Sheagren JN (2001). Increased incidence of *Staphylococcus aureus* bacteremia in hospitalized patients with acquired immunodeficiency syndrome. *Clinical Infectious Diseases*.

[18] Srinivasan A, Seifried S, Zhu L (2009). Short communication: methicillin-resistant *Staphylococcus aureus* infections in children and young adults infected with HIV. *AIDS Research and Human Retroviruses*.

[19] Fergie JE, Purcell K (2001). Community-acquired methicillin-resistant *Staphylococcus aureus* infections in South Texas children. *Pediatric Infectious Disease Journal*.

[20] Pugliese A, Vidotto V, Beltramo T, Torre D (2005). Phagocytic activity in human immunodeficiency virus type 1 infection. *Clinical and Diagnostic Laboratory Immunology*.

